# Type 2 Innate Lymphoid Cells in Liver and Gut: From Current Knowledge to Future Perspectives

**DOI:** 10.3390/ijms20081896

**Published:** 2019-04-17

**Authors:** Aaron Ochel, Gisa Tiegs, Katrin Neumann

**Affiliations:** Institute of Experimental Immunology and Hepatology, Center for Experimental Medicine, University Medical Center Hamburg-Eppendorf, Martinistr. 52, D-20246 Hamburg, Germany; a.ochel@uke.de

**Keywords:** type 2 innate lymphoid cells, gut-liver axis, hepatitis, immune regulation

## Abstract

Innate lymphoid cells (ILCs) represent a heterogeneous population of recently discovered immune cells that mirror the functions of adaptive T lymphocytes. However, ILCs are devoid of specific antigen receptors and cellular activation depends on environmental cytokines, rendering them as early regulators of immune responses. Type 2 innate lymphoid cells (ILC2s) respond to alarmins, such as interleukin-25 and -33 and shape Th2-associated immunity by expressing IL-5 and IL-13 in a GATA3-dependent manner. In addition, ILC2s express the epidermal growth factor-like molecule Amphiregulin thereby promoting regeneration of injured tissue during inflammation. The gut and liver confer nutrient metabolism and bidirectional exchange of products, known as the gut-liver axis. Accordingly, both organs are continuously exposed to a large variety of harmless antigens. This requires avoidance of immunity, which is established by a tolerogenic environment in the gut and liver. However, dysregulations within the one organ are assumed to influence vitality of the other and frequently promote chronic inflammatory settings with poor prognosis. Intensive research within the last years has revealed that ILC2s are involved in acute and chronic inflammatory settings of gut and liver. Here, we highlight the roles of ILC2s in intestinal and hepatic inflammation and discuss a regulatory potential.

## 1. Introduction

Innate lymphoid cells (ILCs) comprise a heterogeneous population of innate immune cells that reflect functional counterparts of adaptive T lymphocytes. ILCs are devoid of known lineage markers, such as CD3, CD11b, B220, Ly6C, Ly6G, and Ly-76 in mice and CD3, CD11c, CD14, CD16, CD19, and CD20 in human. Moreover, ILCs lack rearranged antigen-specific receptors that are required for T cell activation. Depending on the expression of key transcription factors and specific effector molecules, ILCs are categorized into five subsets [1,2,3]. First, natural killer (NK) cells reflect the function of cytotoxic CD8^+^ T lymphocytes and express the transcriptional regulators T-bet and Eomes. Second, group 1 ILCs (ILC1) are T-bet^+^ and mirror the function of CD4^+^ T helper (Th) 1 cells by expressing not only IFN-y, but also granzymes. Thus, NK cells and ILC1s represent important mediators of anti-viral immune responses [4] and they also contribute to chronic intestinal inflammation [5]. Third, ILC2s express the transcription factor GATA3 and are essential mediators of Th2-driven immune responses, such as airway inflammation [6] and expulsion of large extracellular pathogens [7] by expressing IL-5 and IL-13. Next, ILC3s are defined by expression of RORyt and based on the expression of natural cytotoxicity receptor (NCR), are further subdivided into NCR^−^ and NCR^+^ ILC3s that are linked to Th17-associated immunity, such as anti-bacterial [8,9] or anti-fungal [10] defense mechanisms in an IL-17- or IL-22-dependent manner. Finally, RORyt^+^ lymphoid tissue inducer (LTi) cells express lymphotoxin and induce the generation of secondary lymphoid tissue during embryogenesis [11]. Compared to their adaptive counterparts that predominantly home secondary lymphoid tissues, ILCs are clearly underrepresented in lymphoid organs, but reside in various peripheral tissues, such as skin, lung, adipose tissue, intestine, and liver, all of which represent potential entry sides for pathogens. After infection, ILCs are rapidly activated in response to cytokines that are released from the inflamed or injured environment [1]. This in turn, allows to immediately mount effective immune responses by expressing a multitude of pro-inflammatory and immunomodulatory cytokines within hours, while T cell responses depend on clonal expansion and last for several days. Additionally, ILCs fulfill an important role in tissue homeostasis [12], remodeling, and in the repair of injured tissue [13,14,15,16]. This is an important mechanism in response to acute and chronic inflammation. However, chronic inflammation bears the risk of uncontrolled ILC activity thereby aggravating immunopathology [17,18,19,20].

Compared to other peripheral organs, the liver is a primary site of immune induction that is mediated by unique populations of conventional and unconventional antigen-presenting cells (APCs) [21]. The liver exerts nutrient metabolism and detoxification of gut-derived products that are transported through the portal venous blood. The liver establishes a tolerogenic environment to avoid immune responses towards harmless antigens that are metabolized [22]. However, these features predispose the liver as a target for several pathogens, such as hepatitis B and C virus or the malaria sporozoite, which most frequently causes chronic infections. Additionally, loss of tolerance to hepatic autoantigens causes activation of self-reactive T cells that continuously migrate to the liver and induce ongoing necroinflammation and destruction of hepatic parenchyma [23,24]. Consequently, this causes a release of ILC-stimulating cytokines, which promotes the uncontrolled activity of ILCs and aggravation of liver pathology. Indeed, all ILC subsets have been found to be involved in liver diseases [25,26,27,28]. As these findings have already been summarized elsewhere [29], this review will focus on the contribution of ILC2s to the progression of hepatic immune responses. According to the unique immunological properties within the liver and its close connection to the gastrointestinal system, this review will discuss the role of ILC2s during intestinal inflammation and during acute and chronic liver diseases.

## 2. Development, Activation and Regulation of ILC2s

### 2.1. Development

**Figure 1:**
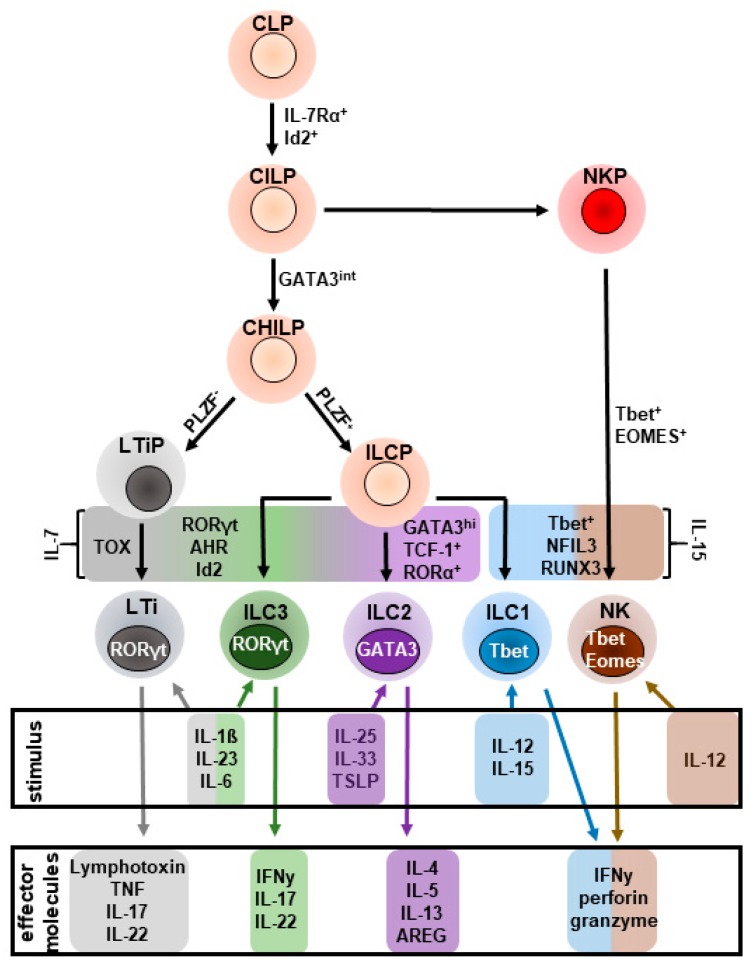
Schematic overview of helper innate lymphoid cell (ILC) development: All helper ILC subsets originate from an IL-7-responsive, inhibitor of DNA binding 2 (Id2)^+^ common lymphoid progenitor (CLP) that further develops into a common innate lymphoid progenitor (CILP). CILP differentiates into an NK cell precursor (NKP) or a common helper innate progenitor (CHILP) that transiently expresses GATA3. The subsequent transcriptional activity of promyelocytic zinc finger (PLZF) further promotes the CHILP to develop into innate lymphoid cell progenitors (ILCP) that differentiate into ILC1-3, while PLZF^−^ CHILP develops into a lymphoid tissue inducer progenitor (LTiP) that further matures into a lymphoid tissue inducer cell (LTi). ILC2-development from the ILCP further requires the expression of the transcription factors T cell factor 1 (TCF-1), RAR-related orphan receptor α (RORα), and GATA binding protein 3 (GATA3). AHR: Aryl hydrocarbon receptor; Eomesodermin; RORγt: RAR-related γt; RUNX3: runt-related transcription factor 3; Tbet: T box transcription factor; TOX: Thymocyte selection-associated high mobility group box protein.

It is widely accepted that all helper ILC subsets originate from an Id2^+^ common helper innate lymphoid precursor (CHILP), located in the fetal liver and adult bone marrow [30]. CHILP is categorized into two subsets based on the expression of promyelocytic leukemia zinc finger (PLZF), which is induced by Id2 [31], and drives the differentiation into ILC1s, ILC2s, and ILC3s, while LTi cells derive from a PLZF^−^ CHILP [30,32]. ILC2 commitment from the PLZF^+^ innate lymphoid cell progenitor (ILCP) [32] requires Notch-dependent induction of the transcriptional regulator TCF-1, which upregulates and stabilizes expression of GATA3 and expression of cytokine receptors, such as interleukin- (IL-)7Rα, IL-17Rb, and IL-2Rα [33,34,35]. As GATA3 is also transiently expressed during ILC1 and ILC3 development [36,37], ILC2 ontogeny from the ILCP requires the concerted action of environmental cytokines, such as IL-7 and IL-33 [38], and the transcriptional regulator RORα [32,33,38,39] (Figure 1). ILC2s express the cytokine receptors IL-7Rα, IL-17Rb, CD25, and the IL-33R, ST2 [19,30,33,34,38,40]. Moreover, ILC2s were demonstrated to express the receptor tyrosine kinase c-Kit, inducible costimulatory (ICOS), and killer cell lectin receptor 1 (KLRG1) [7,33]. All of these regulate ILC2 effector function [41,42]. In addition to ST2^+^ ILC2s, a recent report described a KLRG1^+^ ST2^−^ population of ILC2s, present in the lung, spleen, mesenteric lymph nodes (MLN), and liver, which was dependent on IL-25 for activation. These cells were demonstrated to be early mediators of inflammation during worm infection as they were detectable in the lung of infected mice as early as day 5 post infection, whereas no expansion of ST2^+^ ILC2s was observed at the same time. Accordingly, KLRG1^+^ ST2^−^ ILC2s were termed ‘inflammatory ILC2s’ (iILC2s), while ST2^+^ ILC2s were termed ‘natural ILC2s’ (nILC2s) [43]. Additionally, iILC2s were demonstrated to exert anti-fungal function by acquiring an ILC3-like phenotype [43], which will be discussed in more detail in the following chapters.

### 2.2. Activation

Activation of ILCs occurs in response to specific cytokines that are released from the environmental tissue in any case of perturbation. ILC2s respond quickly to the cytokines IL-25 [43,44], IL-33 [7,40,45], and thymic stromal lymphopoietin (TSLP) [20], but also depend on IL-2 [46]. IL-25 and IL-33 are cytokines with alarmin function that are expressed by barrier cells and released upon cellular necrosis to activate the immune system. TSLP is a member of the IL-7 family that is constitutively expressed in epithelial cells (EC) and keratinocytes [47] and therefore, may facilitate maintenance of ILC2s in the respective tissue. Nevertheless, it appears that activation of ILC2s is individually regulated by the environmental cues of the tissue they populate. For instance, activation of ILC2s that reside within the small intestine seems to require IL-25 derived from intestinal tuft cells upon tissue damage by helminths [48,49]. This assumption is also reflected by the high expression of IL-17Rb on iILC2s and their quick response to *Nippostrongylus brasiliens* compared to intestinal nILC2s [43]. Hepatic ILC2s appear to be mainly dependent on IL-33 [50,51,52], although hepatic expression of IL-25, TSLP, and IL-33 was demonstrated in various inflammatory settings [50,51,53,54,55]. However, it was recently demonstrated that activity of hepatic ILC2s during chronic *Schistosoma mansoni* infection was only ameliorated if IL-25, IL-33, and TSLP were ablated simultaneously, while single interruption had no effect [55]. This suggests that activation of ILC2s underlies a multitude of mediators not only to avoid unspecific activation, but also indicates a redundancy, which allows the establishment of ILC2 responses during inflammation. A similar dependency on multiple activating cytokines was also described for human ILC2s [56], indicating a critical regulatory step in activation of ILC2s across species. Following activation, ILC2s are potent sources of Th2-associated cytokines, such as IL-4, IL-5, IL-9, and IL-13 [35,39,40,56,57]. In addition, ILC2s express the epidermal-like growth factor Amphiregulin (AREG) [14,58] that was initially found to mediate proliferation and survival of non-malignant cells, but to limit the growth of tumor cells [59]. Meanwhile an increasing body of evidence indicates that AREG also favors tumorigenesis [60]. As a growth factor, AREG is crucial for tissue regeneration in lung and liver [14,61]. This licenses ILC2s to regulate tissue homeostasis and maintenance. Moreover, AREG enhances the suppressive function of regulatory T cells (Tregs) [62] and thus, might reflect an immune modulatory role for ILC2s to limit inflammation. However, immune dysregulation and ongoing activation of ILC2s may trigger detrimental changes in tissue architecture and promote organ failure.

### 2.3. Regulation of ILC2s

Apart from the activating cytokines, the activity of ILC2s was demonstrated to be shaped by various surface molecules, neuropeptides, nutrients, and hormones that influence the effector function of ILC2s in a positive or negative manner, respectively (Table 1).

## 3. Immune Regulation in Gut and Liver

According to their anatomical location and function, the gut and the liver are closely connected to each other by both the portal blood and the biliary ducts. This represents a bidirectional interaction between both organs, known as the gut-liver axis. The circulation of blood and bile acids within this axis allows an ongoing exchange of metabolites and it also favors organ vitality.

The gastro-intestinal system is continuously exposed to a large supply of heterogenous antigens that derive from either ingested food or the intestinal microbiota, i.e., the microbiome. The microbiome is segregated from the epithelial cell layer by two intestinal mucus layers that contain mucin Muc2 [75]. The outer layer was demonstrated to contain the intestinal bacteria, while the inner layer forms a physical barrier that impairs direct contact between the bacteria and the endothelium. The microbiome has a crucial part in host physiology, not only as it exerts digestion and metabolism, but also enhances the intestinal barrier. For instance, bacterial production of short chain fatty acids, such as butyrate, was found to be an important regulator to maintain the integrity of the intestinal epithelium [76,77]. The continuous exposure to microbial components requires a delicate balance between immune tolerance and the onset of immunological responses to pathogens to maintain homeostasis. Thus, under homeostatic conditions, the intestinal epithelium establishes a tolerogenic environment, which is mainly attributed to a specialized subset of CD103^+^ intestinal dendritic cells (DCs) [78]. These cells induce production of the anti-inflammatory cytokine IL-10 by Tregs [79] and thereby prevent immune responses to commensal and food-derived antigens [78].

The liver exerts a metabolism of nutrients, carbohydrates, and proteins. It also detoxifies and clears pathogens that derive from the gut and reach the liver by the portal blood [22]. Reciprocally, the vitality of the liver impacts the intestinal function by producing and transporting immunoglobulins, bile acids, and bile salts to maintain the intestinal barrier [80]. Accordingly, the gut-liver axis fulfills a crucial immunological role, as the onset of immune responses to harmless antigens needs to be suppressed in both functionally associated organs. Thus, inflammatory immune responses within the liver are tightly regulated by a unique tolerogenic environment, which is established and maintained by non-parenchymal cells (NPCs) and hepatocytes that constitute the hepatic parenchyma. The major task of hepatocytes is to metabolize nutrients. Even though they present antigens in MHC-I and MHC-II molecules, MHC-I-restricted T cells fail to survive upon hepatocyte-dependent activation, thereby providing functional tolerance [81,82]. In addition, IFN-y expressed during inflammation, upregulates MHC-II expression on hepatocytes and thereby induces IL-10-producing CD4^+^ T cells in a Notch-dependent manner [83]. The non-parenchymal cells (NPCs) inside the liver are sub-divided into liver sinusoidal endothelial cells (LSECs), hepatic stellate cells (HSCs), Kupffer cells (KCs), and intrahepatic DCs. These cells are equipped with MHC-I and MHC-II molecules, but they were shown to be less efficient in T cell priming compared to extrahepatic APCs [84]. This phenomenon is mediated by constitutive expression of immunosuppressive cytokines, such as IL-10, prostaglandin E2 and TGFβ by liver APCs [85], and high expression of the co-inhibitory molecule programmed death ligand (PD-L)1 [86]. Accordingly, the tolerogenic milieu of the liver resembles a suitable niche for various pathogens to establish infections. As outlined above, the liver is closely connected to the gut and disorders within the one environment were shown to also influence the other compartment. Indeed, several chronic inflammatory settings of the liver were recently described to correlate with the breakdown of the intestinal barrier [87]. Reciprocally, the breakdown of the intestinal barrier, due to chronic inflammation might lead to subsequent liver inflammation. For instance, inflammatory bowel disease, is frequently associated with primary sclerosing cholangitis that reflects chronic inflammation of the biliary epithelium within the liver [88]. Thus, it is of tremendous importance for the host to maintain the integrity of the gut-liver axis. On the one hand, this avoids the onset of immune responses. On the other hand, it also overcomes the tolerogenic milieu within the particular organ to ensure that pathogens are eradicated. In contrast to T cells, which become activated by specific antigens and need to undergo clonal expansion, ILC2s are activated by stimulating cytokines. Collectively, these findings indicate that ILCs may react very early and thus shape immune responses in case of perturbation. All ILC subsets are present in the intestine and in the liver and are involved in immune regulation towards viral infection [4,25] and tissue protection [26]. As this review focuses on the role of ILC2s, we will further highlight the function of ILC2s particularly during intestinal and hepatic inflammation.

## 4. ILC2s in Intestinal Inflammation

### 4.1. Intestinal ILC2s Regulate Colitis

**Figure 2:**
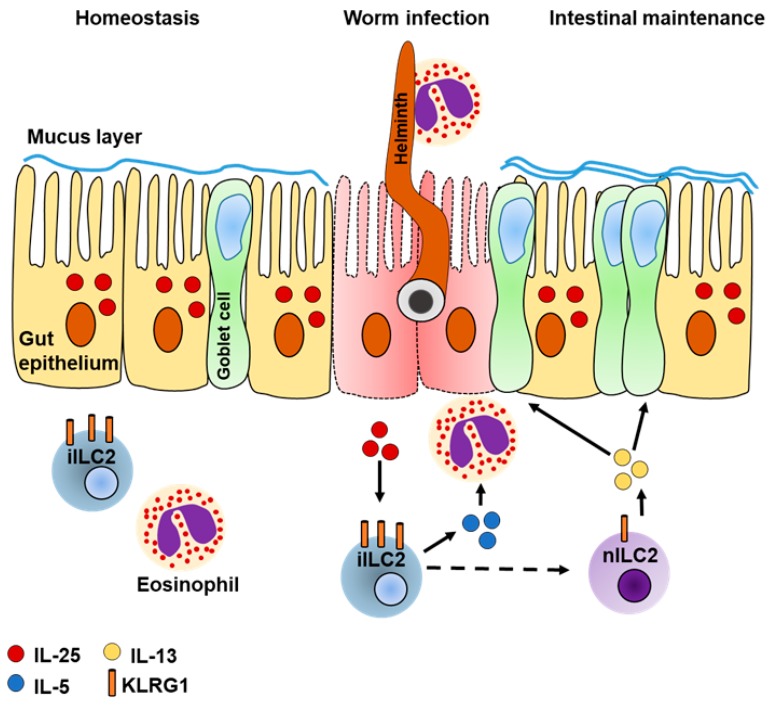
Suggested model of anti-helminth immunity by intestinal inflammatory ILC2s (iILC2s). KLRG1^hi^ iILC2s express IL-5 in response to IL-25-dependent activation to recruit eosinophils that amplify worm expulsion. In parallel, iILC2s convert into natural ILC2s (nILC2s) (indicated by dashed arrow) that downregulate KLRG1 and express IL-13 thereby promoting goblet cell hyperplasia and increased mucus production to restore the function of the intestinal epithelium. Solid arrows indicate the release and effect of the respective stimulus.

Recent studies on murine and human intestinal ILC subsets demonstrated that ILC2s have the lowest abundance compared to ILC1s and ILC3s [54,71,89], but appear to have a significant function during intestinal inflammation. Intestinal ILC2s are mainly described as important mediators to clear helminth infection from the intestine. After activation, intestinal ILC2s express IL-5 to recruit other immune effector cells, such as eosinophils. They also express IL-13, which stimulates mucus production by goblet cells [7,40,45] thereby preventing the breakdown of the intestinal barrier and development of colitis [90,91]. This suggests that intestinal ILC2s do not only contribute to anti-helminth immunity, but also to the maintenance of the intestinal barrier (Figure 2). However, the exact role of ILC2s in the maintenance of the intestine remains controversial. A study using oxazolone to induce colitis in mice recently demonstrated that expression of IL-13 was enhanced in ILC2s in response to IL-25. Colonic inflammation was associated with a significantly reduced colon length. In contrast, ablation of IL-25 in an antibody-dependent manner demonstrated reduced expression of ILC2-derived IL-13 and limited infiltration of Gr1^+^ macrophages. As this in turn correlated with limited disease pathology, it was suggested that IL-25 promotes colitis by subsequent activation of ILC2s [92]. On the other hand, intestinal inflammation in response to dextran sodium sulfate (DSS) revealed an IL-33-dependent expansion of intestinal ILC2s upon tissue damage. Expanded ILC2s expressed AREG and limited intestinal insult as demonstrated by more pronounced disease pathology in AREG-deficient mice [15]. Exogenous application of IL-33 to induce AREG expression in ILC2s, or treatment with AREG itself, caused significant reduction of the disease score, suggesting a tissue-protective role for intestinal ILC2s during colitis [15].

### 4.2. A Potential Role of ILC2-Derived AREG for Intestinal Maintenance

The expression of AREG was recently demonstrated to be an important mediator of ILC2s during wound healing of the injured lung [14] and skin [42] by inducing proliferation of epithelial cells. According to the production of AREG by intestinal ILC2s [15], the capability to express AREG seems to be a general feature of ILC2s to maintain the integrity of the respective tissue. Moreover, as AREG not only induces cellular proliferation, but also enhances the anti-inflammatory potential of Tregs [62], one might speculate that the tissue protective role of ILC2s not only includes regeneration of damaged tissue, but also suppresses the onset of immune responses. This is of particular importance in tolerogenic organs, such as the intestine or the liver, to maintain organ function. However, the exact mechanism explaining how the expression of AREG in ILC2s is regulated remains ill-defined. The regulation of AREG expression within ILC2s was recently demonstrated to be dependent on KLRG1-ligation by E-cadherin on skin-derived ILC2s [42]. Because binding of KLRG1 to E-cadherin reduced the expression of AREG in ILC2s, one might assume a similar regulatory mechanism within the intestine. For instance, E-cadherin itself mediates the assembly and stability of tight junctions within the intestinal epithelium. Loss of these junctions is supposed to affect the epithelial integrity [93]. Thus, one might speculate that during intestinal homeostasis, E-cadherin impairs effector function of intestinal ILC2s, which might be rapidly induced upon loss of E-cadherin to promote effector function of ILC2s and tissue regeneration during intestinal inflammation.

### 4.3. Intestinal ILC2s Are Heterogeneous

In addition to worm expulsion and their somewhat unclear role during colitis, intestinal ILC2s appear to represent a cellular pool of ILC2s that may be distributed systemically in response to infection. This assumption originates from a recent study in parabiotic mice, sharing the blood circulation, due to surgical connection [94]. After helminth infection or intraperitoneal (i.p.) application of IL-25, an enhanced exchange of iILC2s was demonstrated in lungs, MLNs, spleens, and livers of the parabiotic partner, while nILC2s were mainly found to be host specific. Since iILC2s only accumulated in the lungs if IL-25 was applied on the i.p. route, but not intranasally, it was suggested that this was due to cellular migration. Furthermore, adoptive transfers of KLRG1^+^ ILC2s derived from the lung, bone marrow or the small intestine of CD45.1^+^ donors into CD45.2^+^ recipients revealed that only the latter population developed into iILC2s, which was driven in an IL-25-dependent manner [94]. Moreover, IL-25 mediated accumulation of these cells in the lungs of recipient mice, highlighting the high migratory potential of this subset [94]. Additionally, iILC2s were demonstrated to express high levels of α4β7 integrin and the gut-homing receptor CCR9 [94], which was demonstrated to be necessary for ILC2Ps to undergo complete maturation [33]. It might also enable iILC2s to home back to the intestine when infection is cured within the respective organ. However, iILC2s were initially described to constitute an unstable population, as it was found that they developed in ST2^+^ nILC2s, but were also able to acquire an ILC3-like phenotype that was sufficient to confer anti-fungal immunity by expressing IL-17A [43]. All this suggests that iILC2s constitute a premature progenitor population of ILC2s, which has the capability to circulate and to support ILC2- and ILC3-associated immune responses while undergoing complete maturation into the respective subset.

## 5. The Role of ILC2s in Liver Inflammation

### 5.1. Hepatic ILC2s during Acute Liver Inflammation

#### 5.1.1. Role of ILC2s during Immune-Mediated Hepatitis

**Figure 3:**
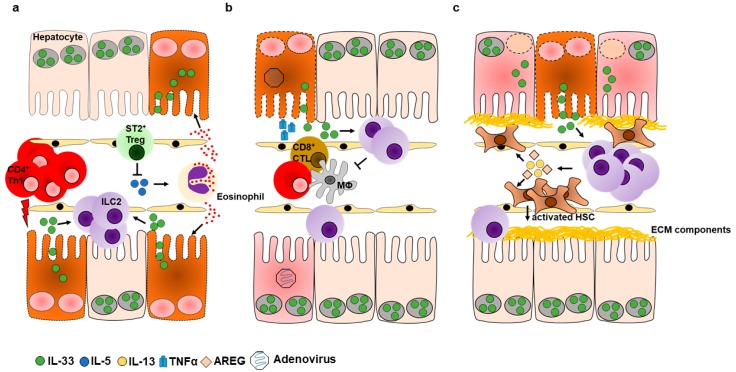
ILC2s have a paradoxical role during acute liver inflammation and promote hepatic fibrogenesis in response to chronic liver injury. (**a**) hepatic ILC2s expand in response to hepatocyte-derived IL-33 during acute immune-mediated hepatitis and amplify inflammation by IL-5-dependent recruitment of eosinophils. Exacerbated necrosis of hepatocytes and ongoing IL-33-release during acute inflammation induces IL-33-responsive Tregs that might limit the activity of ILC2s. (**b**) Adenoviral infection induces TNFα in anti-viral macrophages (MΦ), CD4^+^ Th1 cells, and CD8^+^ cytotoxic T lymphocytes (CTL), thereby promoting the release of IL-33 from dying hepatocytes and activation of ILC2s. Expanded ILC2s limit function of anti-viral immune cells to prevent enhanced loss of hepatic tissue. (**c**) Ongoing necrosis in response to chronic liver injury renders constitutive activation of hepatic ILC2s to release IL-13 and AREG, which activate hepatic stellate cells (HSCs). Following activation, HSCs transform into myofibroblasts that promote liver fibrosis by depositing extra-cellular matrix (ECM) components.

Autoimmune hepatitis (AIH) describes a chronic stage of liver inflammation that is a consequence of loss of tolerance against hepatic auto-antigens. As a result, autoreactive T cells mediate the ongoing destruction of hepatocytes, resulting in severe necroinflammation [24]. The Concanavalin A (Con A) model is a well described murine model to study acute immune-mediated hepatitis in mice [95], and has recently given evidence for a significant contribution of hepatic ILC2s in disease progression [51]. Following CD4^+^ T cell-mediated tissue injury induced by injection of Con A, we showed a rapid release of IL-33 from liver tissue that in turn led to the expansion of hepatic ILC2s that expressed IL-5 and IL-13. As we found amplification, but not induction of immune-mediated hepatitis by ILC2s in response to Con A, the immunogenic role of ILC2s was further investigated by either depletion or adoptive transfer of ILC2s. Accordingly, ILC2 depletion ameliorated liver inflammation, while adoptive transfer of IL-33-elicited hepatic ILC2s exacerbated immune-mediated hepatitis [51]. Additionally, we observed an increased hepatic influx of eosinophils in response to Con A, which was reduced upon depletion of ILC2s, suggesting that ILC2s induce recruitment of eosinophils during immune-mediated hepatitis by expression of IL-5. Indeed, eosinophils were previously demonstrated to amplify disease pathology of immune-mediated hepatitis in an IL-5-dependent manner as a liver injury was reduced upon depletion of either IL-5 or eosinophils [96]. Taken together, our results demonstrate an inflammatory role for ILC2s, which amplify immune-mediated hepatitis by recruitment of eosinophils. This underscores the requirement of innate immune effector cells at early stages of hepatic inflammation to successfully overcome the tolerogenic environment of the liver and thereby contribute to the establishment of potent immune responses (Figure 3a). Of note, exacerbation of immune-mediated hepatitis by IL-33-elicited ILC2s was only seen upon adoptive transfer, whereas mice that were treated with IL-33 prior to Con A challenge were protected from liver inflammation. This was correlated with increased numbers of IL-33-responsive Tregs [51], suggesting a Treg-dependent regulation of ILC2 activity (Figure 3a).

#### 5.1.2. ILC2s Promote Hepatic Protection during Viral Hepatitis

Similar findings of IL-33-mediated protection from liver inflammation were also shown in a model of acute viral hepatitis by adeno-(Ad) infection [27]. Treatment with IL-33 upon Ad-infection resulted in increased expression of Th2-associated cytokines that were correlated with the expansion of hepatic ILC2s and Tregs. Albeit it is well documented that IL-33-elicited Tregs play a critical role in the regulation of liver inflammation [51,97], their contribution in viral hepatitis was not further investigated [27]. Furthermore, it was found that IL-33 treatment abrogated TNFα production in CD4^+^ and CD8^+^ T lymphocytes, as well as in CD11b^+^ cells, which reflects a critical feature of Tregs. However, co-cultivation of freshly isolated lineage^−^ cells with CD4^+^, CD8^+^ or CD11b^+^ cells derived from Ad-infected mice in presence of ILC2-activating cytokines (IL-2, IL-7, and IL-33) demonstrated reduced TNFα production in the respective population of immune effector cells. Thus, it was suggested that ILC2s limit liver pathology during viral infection by suppressing Th1-associated immune responses (Figure 3b). However, adoptive transfer of activated ILC2s to Ad-infected mice resulted only in the mild improvement of liver pathology. Hence, it was concluded that the IL-33-mediated protection from liver inflammation was due to a complex network of IL-33-responsive cells that cooperate during tissue protection [27]. Indeed, it was recently demonstrated that activated ILC2s contribute to tissue protection in an indirect fashion by the polarization of alternatively activated macrophages (AAMs). In a model of cerebral malaria, it was recently shown that ILC2-primed AAMs were required to induce Tregs in an unknown mechanism, which were found to limit disease pathology in response to IL-33 [98]. Thus, one might speculate that the results obtained from Liang et al., are due to initial activation of ILC2s, which subsequently induce other immune regulatory cells that mediate protection from liver injury during viral hepatitis.

Taken together, ILC2s appear to be involved in the regulation of different types of acute hepatic inflammation. Even though both experimental settings described depend on T cell-mediated liver injury, due to the expression of TNFα it must be noted that both models underlie different kinetics. The Con A-induced hepatitis model is a very acute model that depends on pan-T cell activation and results in immune-mediated hepatitis within a few hours, whereas anti-viral responses require the classical course of immunity, namely antigen presentation, T cell activation and clonal expansion. As transfers of IL-33-elicited ILC2s in the latter study were performed on days +1, +3, and +5 post infection, i.e., time points when the viral infection is already established, the particular role of ILC2s at the time point, when the virus targets the liver remains elusive. In addition, despite the reduction of TNFα observed in the immune-effector cells during co-cultures, it is worth noting that the ILC2s in this experimental setting derived from uninfected donors that were treated with IL-33.

#### 5.1.3. Plasticity of ILC2s

A recent study on human airway ILC2s demonstrated a retinoic acid (RA)-dependent conversion of ILC2s into a regulatory phenotype (ILCregs) [73]. ILCregs were devoid of CRTH-2 and ST2 and they downregulated ILC2 signature cytokine expression. Instead, they were found to express Treg-associated surface molecules, such as cytotoxic T-lymphocyte associated protein 4 (CTLA-4) and CD25. Furthermore, ILCregs expressed the anti-inflammatory cytokine IL-10, and suppressed CD4^+^ T cells and ILC2s. If comparing the abundance of ILCregs between healthy controls and patients with chronic rhinosinusitis, they were found to be low in the former group, but increased in nasal tissues in the latter. With regard to the plastic potential of iILC2s, discussed in chapter 4.3, these data suggest that ILC2s represent a heterogenous sub-population that is capable to adjust to the inflammatory environment and thereby represents biological significance in immune-induction, but also –regulation.

A recent study on the function and phenotype of murine airway ILC2s described their capability to acquire a memory-like phenotype, that allows a rapid reactivation of ILC2s in response to reoccurring stimuli [99]. Following pre-activation of airway ILC2s by either IL-33 or the allergen-protease papain (PAP) on three consecutive days, numbers of ILC2s remained high in either condition for more than four weeks and showed low levels of proliferation, as evidenced by BrdU-incorporation. Interestingly, the persisting cells not only responded to their initial stimulus, but also to an unrelated antigen as shown by pre-activation with the fungal allergen *Aspergillus oryzae* serine protease and reactivation with PAP [99]. Finally, comparative gene expression profiling of naïve and IL-33-elicited ILC2s on day 4, week 2, or four months upon initial IL-33-application revealed that the expressed genes were comparable to that of naïve and memory T cells. This suggests that even though ILC2s are members of the innate immune system, they are able to constitute a memory population that might contribute to ongoing disease pathology. Compared to effector ILC2s, it was found that memory ILC2s upregulated the IL-25R, and indeed responded to IL-25, unlike naïve ILC2s [99]. Thus, memory ILC2s can not only react to the same reoccurring stimulus, according to the common dogma regarding adaptive memory cells, but also to other stimuli. However, as also true for memory cells of the adaptive immune system, the activation threshold of memory ILC2s was found to be lower, as these cells also reacted to single injections of stimuli [99].

### 5.2. Hepatic ILC2s in Chronic Liver Inflammation

Chronic inflammation is a consequence of continuous tissue destruction in response to the failure of efficient pathogen elimination, autoimmune disorders or cellular stress. It is therefore characterized by an ongoing turnover of cellular death and regeneration of injured tissue. Hence, chronic inflammation is a pre-requisite of liver fibrosis that progresses towards liver cirrhosis and hepatocellular carcinoma (HCC) [100]. IL-33 was recently found to be a prognostic marker of liver inflammation, as the serum levels were elevated in patients with acute and chronic hepatitis B virus infection and in patients with liver cirrhosis, but they were low in healthy controls [50,51]. The IL-33-dependent progression of liver fibrosis was recently demonstrated during chronic liver inflammation in mice [50]. Repeated application of carbon-tetrachloride (CCl_4_) is a suitable tool to induce chronic liver inflammation and showed the elevated release of IL-33 that correlated with expansion and activation of hepatic ILC2s. Activated ILC2s expressed IL-13 that subsequently triggered trans-differentiation of HSCs into myofibroblasts that produced extra-cellular matrix (ECM)-components thereby promoting liver fibrosis. Since it was observed that ECM deposition and thereby fibrogenesis were reduced in mice devoid of either IL-33- or IL-13-signalling, it was concluded that liver fibrosis was solely dependent on activated ILC2s [50]. These data reveal a profibrotic function of hepatic ILC2s in chronically inflamed liver tissue. A similar role for ILC2s was seen in studies on human liver explants that were taken at an end-stage of severe liver disease [53,54]. While both studies confirmed the expression of IL-13 by ILC2s, the former also showed a high frequency of AREG expressing ILC2s in both non-inflamed and diseased hepatic tissue derived from patients with end-stage liver disease [53]. This suggests that ILC2-derived AREG is also involved in liver fibrosis and cirrhosis. Indeed, AREG was found to be a crucial regulator of hepatic regeneration in response to acute liver injury [61]. Moreover, AREG was found to induce α-smooth muscle actin and collagen in murine livers following chronic inflammation by CCl_4_ [101], and to drive hepatic fibrogenesis by activating hepatic stellate cells (HSCs) during non-alcoholic steatohepatitis [102] (Figure 3c). In conclusion, in mice and humans hepatic ILC2s are essentially involved in the pathogenesis of hepatic fibrosis by expressing IL-13. However, it remains elusive whether liver fibrosis is also dependent on other ILC2-derived factors, such as AREG. As discussed in the previous chapter, it appears that hepatic ILC2s have a bivalent role in hepatic inflammation. Liver fibrosis is a consequence of chronic inflammation, in which cellular necrosis and subsequent regeneration is an ongoing process. Compared to acute inflammation, the cellular necrosis and thus, the release of IL-33 is lower in chronic inflammation. Therefore, the function of ILC2s might be shaped by the particular inflammatory condition. On the one hand, strong cellular necrosis and, thus, a strong release of IL-33 might induce a pro-inflammatory function in ILC2s that cooperate with eosinophils to enhance acute liver inflammation. On the other hand, ongoing levels of cell death during chronic inflammation might shape a repair phenotype that activates HSCs and thus, promotes fibrogenesis.

### 5.3. Liver Cancer

**Figure 4:**
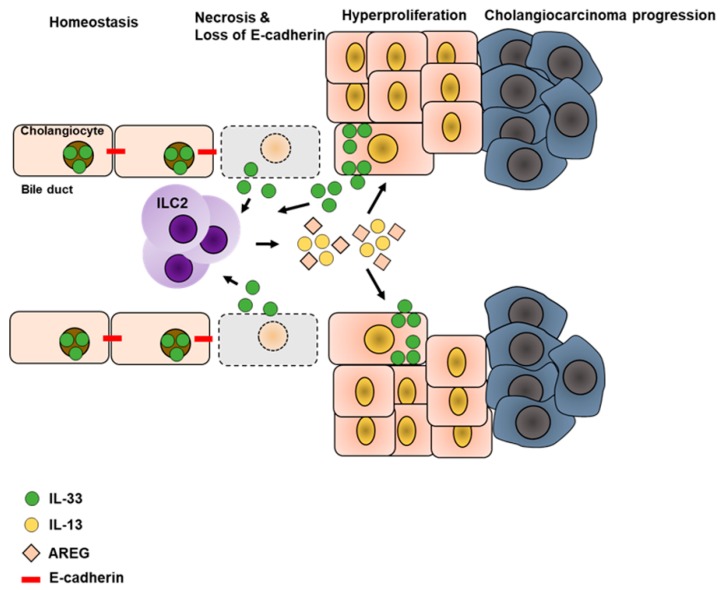
Hepatic ILC2s contribute to the progression of cholangiocarcinoma in extra hepatic bile ducts. Loss of E-cadherin on cholangiocytes and enhanced cellular necrosis favor activation of hepatic ILC2s in an IL-33-dependent manner, thereby promoting expression of IL-13 and AREG. IL-13 and AREG induce hyperproliferation in cholangiocytes and enhanced expression of IL-33, inducing the ongoing activity of ILC2s that favor malignant transformation and progression of cholangiocarcinoma.

Disturbance in the biliary epithelium lining the hepatic bile ducts (also known as cholangiocytes) is frequently observed in response to chronic liver inflammation, and progress towards hepatic fibrosis and cholangiocyte carcinoma (CC). Hyperplasia of cholangiocytes is a critical step in CC formation and it was recently found that IL-33 drives cholangiocyte proliferation as confirmed by enhanced incorporation of BrdU [13]. Further analysis revealed that this enhanced proliferation was in fact triggered by ILC2-derived IL-13, which was required to promote cholangiocyte repair and cholangiocyte hyperplasia in response to IL-33. This indicates a carcinogenic role for IL-33-dependent ILC2s in the chronic setting of liver inflammation. Carcinogenesis is a multi-facetted process established by malignant cells that lost control of the cell cycle and underwent neoplastic transformation [103]. CC is frequently associated with the cellular transformation of cholangiocytes, which downregulate cellular adhesion molecules and disseminate from the epithelial cell layer. Using a model of CC formation and progression [52,104], it was recently shown that additional loss of E-cadherin favors CC development in an IL-33-dependent fashion [52]. Disruption of the E-cadherin encoding gene enhanced expression of IL-33 in biliary epithelial cells, which was excessively released during cellular necrosis and also upon successful establishment of CC. Accordingly, the authors observed elevated amounts of activated ILC2s in the bile ducts, which expressed IL-5, IL-13, and AREG. As AREG induced not only proliferation of biliary epithelial cells in this mouse model, but also of cells derived from human CC, it was concluded that constitutive activation of ILC2s and release of IL-13, but mainly AREG, promotes biliary tumors [52] (Figure 4). In line with this, elevated expression of AREG was found in a variety of tumors [60,105,106] and according to its involvement in liver regeneration during acute and chronic liver inflammation, one might speculate that AREG plays an essential role in the development of HCC. Moreover, beyond its regenerative potential, AREG was also demonstrated as an anti-inflammatory molecule that limits pathology in various inflammatory conditions [16] and also enhances the suppressive function of Tregs [62,107]. The sum of these features suggest AREG as an important pro-tumorigenic molecule during unresolved chronic inflammation as it might: (1) Promote continuous regeneration of wounded tissue thereby promoting fibrosis and cirrhosis; and (2) suppress immune responses to malignant cells by enhancing suppressive function of Tregs, as recently demonstrated in a B16-F10 tumor model [62]. With regard to hepatic ILC2s, dysregulated activation in response to ongoing tissue injury and IL-33 release indicates a carcinogenic role for ILC2s by continuous AREG expression that in turn favors neoplastic transformation of the epithelium and establishes immunosuppressive conditions by promoting Tregs. Thus, understanding the pathways, which regulate ILC2s and also the interaction of ILC2s and Tregs are of utmost importance.

In summary, ILC2s are essential immunogenic mediators during acute liver inflammation and infection. Furthermore, ILC2s also have a significant role during chronic liver disease as they are linked to the progression of liver fibrosis and therefore might also promote the development of liver cancer in response to liver fibrosis. As these effects of ILC2s are mediated by IL-13 and AREG expression, induced in response to IL-33, these molecules represent potential targets to treat fibrotic patients and to prevent cancer progression.

However, the role of ILC2-derived AREG during acute and chronic stages of hepatic inflammation remains to be defined sufficiently. Even though hepatic ILC2s were demonstrated to express AREG [53], there is not yet any current evidence that ILC2s contribute to immune regulation or tissue regeneration by releasing AREG. Additionally, the pathways that modulate the activity of hepatic ILC2s remain elusive. Thus, a deeper understanding of hepatic ILC2s function during acute and chronic liver inflammation is required.

## 6. Potential Migration of ILC2s

With the exception of iILC2s, ILCs in general are regarded as tissue-resident cells. This assumption comes from the finding that ILCs were equally distributed in various tissues during homeostasis. However, as their frequencies were found to increase rapidly upon infection, it was suggested that ILCs might either have a rapid transition from the blood into the tissue or undergo local expansion during infection [45]. Similar results were obtained by initial studies in parabiotic mice because only little to no exchange of ILCs was found to take place during homeostasis or acute inflammation. In contrast, NK cells and T lymphocytes were equally distributed between surgically connected partners [108,109]. Although low migration of ILC2s occurred during chronic stages of *N. brasiliensis* infection, the majority of ILCs were derived from the respective host [108]. Thus, it was suggested that migration of ILCs is only valid if prolonged inflammatory conditions require additional effector cells. However, based on the migratory potential of intestinal iILC2s as discussed above and on the continuous exchange via the gut-liver-axis, one might speculate that there is also a continuous exchange of iILC2s. Reciprocally, such an exchange might explain the close correlation between intestinal and liver inflammation. Since iILC2s were demonstrated to develop into nILC2s [43], iILC2s might become activated during intestinal inflammation and subsequently migrate to the liver. Once they reach the liver, iILC2s develop into nILC2s, but continue to express IL-5 and IL-13, thereby recruiting eosinophils and triggering liver pathology. As this in turn, favors release of IL-33, there might be an ongoing activation of ILC2s, which consequently promotes liver fibrosis and cirrhosis and thereby reciprocally impacts the vitality of the gut. Additionally, with regard to the conversion of ILC2s into ILCregs mediated by RA, ILC2s might also be involved in maintaining the tolerogenic environment of the gut. This assumption is supported by a recent report on nutrient-dependency for ILCs [71]. It was observed that intestinal ILC2s expanded during vitamin A deficiency (which is the non-metabolized form of RA) and expressed IL-13, but were reduced in the presence of vitamin A. Simultaneously, intestinal ILC3s were found to be highly abundant when vitamin A was present. As vitamin A deficiency is prone to enhance susceptibility for bacterial infection, it was proposed that this balance of ILC2s vs. ILC3s is a regulative mechanism to maintain intestinal barrier function in a vitamin A-dependent manner [71]. Mechanistically, it was found that ILC2s downregulate the expression of IL-7Rα in the presence of vitamin A thereby providing a benefit for ILC3s in the competition of growth factors. However, this study does not include the possibility that ILC2s were converted into ILCregs in the presence of vitamin A and as such decreased frequencies of intestinal ILC2s might be due to a completely converted phenotype. Furthermore, it might represent a potential regulatory mechanism for ILC2s to maintain the tolerogenic environment of the intestine. In this suggested scenario, vitamin A might promote conversion of intestinal ILC2s into IL-10-producing ILCreg, which contribute towards establishing intestinal tolerance. However, since ILCregs are the most recently identified subset of ILCs (and they, as of yet, have only been described in the airways), understanding these cells will require a deeper characterization in various tissues, and investigation that might help to understand whether these cells could also convert back into ILC2s.

## 7. Summary

Even though ILCs constitute the most recently identified population of immune effector cells, the intensive research on these immune cells has uncovered tremendous information within the last decade allowing us to understand the biology and function of ILCs. Based on their location on barrier sites and their capability to quickly respond to any given disturbance within the respective tissue, ILCs appear to represent essential mediators of organ integrity. While all ILC subsets are important to confer defense against pathogens, ILC2s seem to be the most important for tissue repair. ILC1s and ILC3s support the killing of infected cells or protection of intact tissue. We have summarized the contribution of hepatic and intestinal ILC2s during acute and chronic inflammation and highlighted their potential to be quick responders during acute inflammation. Yet, as with virtually every immune cell, uncontrolled activity of ILC2s during chronic inflammation contributes to changes within the environment, and has tremendous effects on liver architecture and function. In turn, this might also impact the vitality of the intestine. Thus, it is important to further dissect the mechanisms, which might be responsible for limiting the uncontrolled activity of ILC2s and also restoring organ function.

In addition, the role of human ILC2s in inflammatory settings remains to be understood in more detail. The research conducted in mice significantly contributed to the principle understanding of hepatic and intestinal ILC2s during inflammation and maintenance of tissue function. However, the findings regarding the role of hepatic and intestinal ILC2s in humans are as of yet very limited and there is thus a tremendous need to further characterize these cells in the diseased human setting.

## 8. Perspectives

Liver inflammation is frequently diagnosed at late stages, after chronic liver injury has already been established. IL-33 appears to represent a valuable biomarker of tissue injury and the ongoing activity of ILC2s is triggered by IL-33, targeting either IL-33 or ILC2-released cytokines may provide new therapeutic approaches to limit fibrosis and cirrhosis. As IL-13 has already been identified as a driver of liver fibrosis, it remains unclear whether ILC2-derived AREG also influences disease progression. Although the fibrotic potential of AREG has already been demonstrated, its cellular sources remain elusive. However, as hepatic ILC2s from end-stage livers were demonstrated to express AREG, the contribution of this molecule should be further investigated. Moreover, the exact mechanism that promotes an ongoing activity of ILC2s in response to continuous IL-33 exposure remains elusive. Since repeated application of IL-33 enhances regulatory Tregs that are known regulators of liver inflammation [110], these cells are suggested to confer IL-33-mediated protection against acute hepatitis by limiting the function of pro-inflammatory cells, including ILC2s. Tregs were recently demonstrated to directly regulate the function of ILC2s in an ICOS-ICOSL-dependent manner, resulting in limited ILC2-driven asthma [65]. Moreover, ICOS signaling was already demonstrated to be involved in the progression of immune-mediated hepatitis, as liver inflammation was dampened in ICOS-deficient mice following Con A treatment [111]. Even though hepatic ILC2s were not investigated in this report, it still highlights the significance of ICOS signaling in general. This might bear a potential target to limit the activity of ILC2s, as their maintenance was shown to be dependent on ICOS [63,64]. However, with regard to the finding that Tregs limited activity of ILC2s, blocking of ICOS might bear the risk also to lower the activity of Tregs and other effector cells. Additionally, ILC2s are regulated by integrated signals that derive from the positive regulator KLRG1 or the negative regulator PD-1. The ligand of PD-1, PD-L1, is highly expressed within the homeostatic liver. Moreover, E-cadherin that limits KLRG1 signaling was shown to be involved in maintaining the hepatic parenchyma. This indicates that the activity of hepatic ILC2s might be impaired by multiple signaling pathways in the healthy liver, which are dysregulated during inflammation. In conclusion, while there is an increasing body of evidence indicating the contribution of ILC2s in acute and chronic liver inflammation, finding possible interventions that specifically impair the ongoing activity of ILC2s will require further functional studies.

## Figures and Tables

**Table 1:** Overview of positive and negative regulators for ILC2 activity. Even though the regulators listed here were well described for lung, skin and intestinal ILC2s, the regulatory pathways for hepatic ILC2s remain elusive. Thus, this table summarizes known regulatory mechanisms that might also modulate the function of hepatic ILC2s.

Molecule	Function	Expressed on	Demonstrated on	References
ICOS	Maturation and maintenance	ILC2s	murine and human ILC2s	[63,64]
ICOS-L	Negative regulator of ILC2 activity by ligation of ICOS^+^ iTregs	ILC2s	murine ILC2s	[65]
KLRG1	Regulates phosphorylation of STAT5 to enhance proliferation and effector cytokine production	ILC2s	murine and human ILC2s	[41,42]
E-cadherin	Impairs ILC2 function by ligation of KLRG1	Epithelial cells	human ILC2s	[42]
Programmed death (PD)-1	Negative regulator of KLRG1 signaling, impairs phosphorylation of STAT5	ILC2s	murine and human ILC2s	[41]
**Neuropeptides**				
Calcitonin gene-related peptide (CGRP)	Amplifies effector of lung ILC2s		murine ILC2s	[66]
Neuromedin U (NMU)	Induces ILC2 effector function		murine ILC2s	[67,68,69]
Vasoactive intestinal peptide (VIP)	regulates IL-5 production in ILC2s, confers tissue homeostasis by eosinophil-recruitment		murine ILC2s	[70]
**Nutrients**				
Retinoic acid (RA)	Downregulates expression of IL-7Rα and thus, limits cellular maintenance of murine ILC2s.		murine ILC2s	[71]
	Enhances expression of ILC2-derived effector cytokines on human ILC2s.		human ILC2s	[72]
	converts ILC2s into a regulatory phenotype (ILC2reg)		human ILC2s	[73]
**Hormones**				
Androgens	Negative regulator of ILC2 effector function		murine ILC2s	[74]

## References

[1] Vivier E., Artis D., Colonna M., Diefenbach A., Di Santo J.P., Eberl G., Koyasu S., Locksley R.M., McKenzie A.N.J., Mebius R.E. (2018). Innate Lymphoid Cells: 10 Years On. Cell.

[2] Sonnenberg G.F., Artis D. (2015). Innate lymphoid cells in the initiation, regulation and resolution of inflammation. Nat. Med..

[3] Klose C.S.N., Artis D. (2016). Innate lymphoid cells as regulators of immunity, inflammation and tissue homeostasis. Nat. Immunol..

[4] Weizman O.-E., Adams N.M., Schuster I.S., Krishna C., Pritykin Y., Lau C., Degli-Esposti M.A., Leslie C.S., Sun J.C., O’Sullivan T.E. (2017). ILC1 confer early host protection at initial sites of viral infection. Cell.

[5] Bernink J.H., Peters C.P., Munneke M., Velde A.A.T., Meijer S.L., Weijer K., Hreggvidsdottir H.S., Heinsbroek E.S., Legrand N., Buskens C.J. (2013). Human type 1 innate lymphoid cells accumulate in inflamed mucosal tissues. Nat. Immunol..

[6] Morita H., Arae K., Unno H., Miyauchi K., Toyama S., Nambu A., Oboki K., Ohno T., Motomura K., Matsuda A. (2015). An Interleukin-33-Mast Cell-Interleukin-2 Axis Suppresses Papain-Induced Allergic Inflammation by Promoting Regulatory T Cell Numbers. Immunity.

[7] Neill D.R., Wong S.H., Bellosi A., Flynn R.J., Daly M., Langford T.K.A., Bucks C., Kane C.M., Fallon P.G., Pannell R. (2010). Nuocytes represent a new innate effector leukocyte that mediates type-2 immunity. Nature.

[8] Van Maele L., Carnoy C., Cayet D., Ivanov S., Porte R., Deruy E., Chabalgoity J.A., Renauld J.-C., Eberl G., Benecke A.G. (2014). Activation of Type 3 Innate Lymphoid Cells and Interleukin 22 Secretion in the Lungs During Streptococcus pneumoniae Infection. J. Infect. Dis..

[9] Satoh-Takayama N., Vosshenrich C.A.J., Lesjean-Pottier S., Sawa S., Lochner M., Rattis F., Mention J.J., Thiam K., Cerf-Bensussan N., Mandelboim O. (2008). Microbial Flora Drives Interleukin 22 Production in Intestinal NKp46+ Cells that Provide Innate Mucosal Immune Defense. Immunity.

[10] Gladiator A., Wangler N., Trautwein-Weidner K., LeibundGut-Landmann S. (2013). Cutting Edge: IL-17-Secreting Innate Lymphoid Cells Are Essential for Host Defense against Fungal Infection. J. Immunol..

[11] Mebius R.E., Rennert P., Weissman I.L. (1997). Developing lymph nodes collect CD4+CD3-LTβ+cells that can differentiate to APC, NK cells, and follicular cells but not T or B cells. Immunity.

[12] Aparicio-Domingo P., Romera-Hernandez M., Karrich J.J., Cornelissen F., Papazian N., Lindenbergh-Kortleve D.J., Butler J.A., Boon L., Coles M.C., Samsom J.N. (2015). Type 3 innate lymphoid cells maintain intestinal epithelial stem cells after tissue damage. J. Exp. Med..

[13] Li J., Razumilava N., Gores G.J., Walters S., Mizuochi T., Mourya R., Bessho K., Wang Y.-H., Glaser S.S., Shivakumar P. (2014). Biliary repair and carcinogenesis are mediated by IL-33–dependent cholangiocyte proliferation. J. Clin. Investig..

[14] Monticelli L.A., Artis D. (2012). Innate lymphoid cells promote lung tissue homeostasis following acute influenza virus infection. Nat. Immunol..

[15] Monticelli L.A., Osborne L.C., Noti M., Tran S.V., Zaiss D.M.W., Artis D. (2015). IL-33 promotes an innate immune pathway of intestinal tissue protection dependent on amphiregulin—EGFR interactions. Proc. Natl. Acad. Sci. USA.

[16] Zaiss D.M., Gause W.C., Osborne L.C., Artis D. (2015). Emerging functions of amphiregulin in orchestrating immunity, inflammation and tissue repair. Immunity.

[17] Chang Y.-J., Kim H.Y., Albacker A.L., Baumgarth N., McKenzie A.N.J., Smith E.D., DeKruyff R.H., Umetsu D.T. (2011). Innate lymphoid cells mediate influenza-induced airway hyper-reactivity independently of adaptive immunity. Nat. Immunol..

[18] Buonocore S., Ahern P.P., Uhlig H.H., Ivanov I.I., Littman D.R., Maloy K.J., Powrie F. (2010). Innate lymphoid cells drive interleukin-23-dependent innate intestinal pathology. Nature.

[19] Halim T.Y., Krauß R.H., Sun A.C., Takei F. (2012). Lung Natural Helper Cells Are a Critical Source of Th2 Cell-Type Cytokines in Protease Allergen-Induced Airway Inflammation. Immunity.

[20] Kim B.S., Siracusa M.C., Saenz S.A., Noti M., Monticelli L.A., Sonnenberg G.F., Hepworth M.R., Van Voorhees A.S., Comeau M.R., Artis D. (2013). TSLP elicits IL-33–independent innate lymphoid cell responses to promote skin inflammation. Sci. Transl. Med..

[21] Horst A.K., Neumann K., Diehl L., Tiegs G. (2016). Modulation of liver tolerance by conventional and nonconventional antigen-presenting cells and regulatory immune cells. Cell. Mol. Immunol..

[22] Protzer U., Maini M.K., Knolle P. (2012). A Living in the liver: Hepatic infections. Nat. Rev. Immunol..

[23] Sebode M., Hartl J., Vergani D., Lohse A.W., The International Autoimmune Hepatitis Group (IAIHG) (2017). Autoimmune hepatitis: From current knowledge and clinical practice to future research agenda. Liver Int..

[24] Manns M.P., Lohse A.W., Vergani D. (2015). Autoimmune hepatitis—Update 2015. J. Hepatol..

[25] Tang T., Yang Z., Wei X., Yang S., Tian Z. (2015). Type 1 innate lymphoid cells contribute to the pathogenesis of chronic hepatitis B. Innate Immun..

[26] Matsumoto A., Kanai T., Mikami Y., Chu P.S., Nakamoto N., Ebinuma H., Saito H., Sato T., Yagita H., Hibi T. (2013). IL-22-Producing RORγt-Dependent Innate Lymphoid Cells Play a Novel Protective Role in Murine Acute Hepatitis. PLoS ONE.

[27] Liang Y., Jie Z., Hou L., Aguilar-Valenzuela R., Vu D., Soong L., Sun J. (2013). IL-33 induces nuocytes and modulates liver injury in viral hepatitis. J. Immunol..

[28] Jie Z., Liang Y., Hou L., Dong C., Iwakura Y., Soong L., Cong Y., Sun J. (2014). Intrahepatic Innate Lymphoid Cells Secrete IL-17A and IL-17F That Are Crucial for T Cell Priming in Viral Infection. J. Immunol..

[29] Liu M., Zhang C. (2017). The Role of Innate Lymphoid Cells in Immune-Mediated Liver Diseases. Front. Immunol..

[30] Klose C.S., Flach M., Möhle L., Rogell L., Hoyler T., Ebert K., Fabiunke C., Pfeifer D., Sexl V., Fonseca-Pereira D. (2014). Differentiation of Type 1 ILCs from a Common Progenitor to All Helper-like Innate Lymphoid Cell Lineages. Cell.

[31] Verykokakis M., Krishnamoorthy V., Iavarone A., Lasorella A., Sigvardsson M., Kee B.L. (2013). Essential functions for ID proteins at multiple checkpoints in invariant NKT cell development. J. Immunol..

[32] Constantinides M.G., McDonald B.D., Verhoef P.A., Bendelac A. (2014). A committed precursor to innate lymphoid cells. Nature.

[33] Hoyler T., Klose C.S., Souabni A., Turqueti-Neves A., Pfeifer D., Rawlins E.L., Voehringer D., Busslinger M., Diefenbach A. (2012). The Transcription Factor GATA-3 Controls Cell Fate and Maintenance of Type 2 Innate Lymphoid Cells. Immunity.

[34] Yang Q., Monticelli L.A., Saenz S.A., Chi A.W.S., Sonnenberg G.F., Tang J., De Obaldia M.E., Bailis W., Bryson J.L., Toscano K. (2013). T Cell Factor 1 Is Required for Group 2 Innate Lymphoid Cell Generation. Immunity.

[35] Mjösberg J., Bernink J., Golebski K., Karrich J.J., Peters C.P., Blom B., Velde A.A.T., Fokkens W.J., Van Drunen C.M., Spits H. (2012). The Transcription Factor GATA3 Is Essential for the Function of Human Type 2 Innate Lymphoid Cells. Immunity.

[36] Serafini N., Klein Wolterink R.G.J., Satoh-Takayama N., Xu W., Vosshenrich C.A.J., Hendriks R.W., Di Santo J.P. (2014). Gata3 drives development of RORγt + group 3 innate lymphoid cells. J. Exp. Med..

[37] Yagi R., Zhong C., Northrup D.L., Yu F., Bouladoux N., Spencer S., Hu G., Barron L., Sharma S., Nakayama T. (2014). The transcription factor GATA3 is critical for the development of all IL-7Rα-expressing innate lymphoid cells. Immunity.

[38] Wong S.H., Walker J.A., Jolin H.E., Drynan L.F., Hams E., Camelo A., Barlow J.L., Neill D.R., Panova V., Koch U. (2012). Transcription factor RORα is critical for nuocyte development. Nat. Immunol..

[39] Halim T.Y., MacLaren A., Romanish M.T., Gold M.J., McNagny K.M., Takei F. (2012). Retinoic-Acid-Receptor-Related Orphan Nuclear Receptor Alpha Is Required for Natural Helper Cell Development and Allergic Inflammation. Immunity.

[40] Moro K., Yamada T., Tanabe M., Takeuchi T., Ikawa T., Kawamoto H., Furusawa J.I., Ohtani M., Fujii H., Koyasu S. (2010). Innate production of TH2 cytokines by adipose tissue-associated c-Kit+Sca-1+lymphoid cells. Nature.

[41] Taylor S., Huang Y., Mallett G., Stathopoulou C., Felizardo T.C., Sun M.-A., Martin E.L., Zhu N., Woodward E.L., Elias M.S. (2017). PD-1 regulates KLRG1 + group 2 innate lymphoid cells. J. Exp. Med..

[42] Salimi M., Barlow J.L., Saunders S.P., Xue L., Gutowska-Owsiak D., Wang X., Huang L.-C., Johnson D., Scanlon S.T., McKenzie A.N. (2013). A role for IL-25 and IL-33–driven type-2 innate lymphoid cells in atopic dermatitis. J. Exp. Med..

[43] Huang Y., Guo L., Qiu J., Chen X., Hu-Li J., Siebenlist U., Williamson P.R., Urban J.F., E Paul W. (2014). IL-25-responsive, lineage-negative KLRG1hi cells are multipotential ‘inflammatory’ type 2 innate lymphoid cells. Nat. Immunol..

[44] Saenz S.A., Noti M., Artis D. (2010). Innate immune cell populations function as initiators and effectors in Th2 cytokine responses. Trends Immunol..

[45] Price A.E., Liang H.-E., Sullivan B.M., Reinhardt R.L., Eisley C.J., Erle D.J., Locksley R.M. (2010). Systemically dispersed innate IL-13–expressing cells in type 2 immunity. Proc. Natl. Acad. Sci. USA.

[46] Mirchandani A.S., Besnard A.-G., Yip E., Scott C., Bain C.C., Cerovic V., Salmond R.J., Liew F.Y. (2014). Type 2 Innate Lymphoid Cells Drive CD4+ Th2 Cell Responses. J. Immunol..

[47] Takai T. (2012). TSLP Expression: Cellular Sources, Triggers, and Regulatory Mechanisms. Allergol. Int..

[48] Howitt M.R., Lavoie S., Michaud M., Blum A.M., Tran S.V., Weinstock J.V., Gallini C.A., Redding K., Margolskee R.F., Osborne L.C. (2016). Tuft cells, taste-chemosensory cells, orchestrate parasite type 2 immunity in the gut. Science.

[49] Gerbe F., Sidot E., Smyth D.J., Ohmoto M., Matsumoto I., Dardalhon V., Cesses P., Garnier L., Pouzolles M., Brulin B. (2016). Intestinal epithelial tuft cells initiate type 2 mucosal immunity to helminth parasites. Nature.

[50] Mchedlidze T., Waldner M., Zopf S., Walker J., Rankin A.L., Schuchmann M., Voehringer D., McKenzie A.N.J., Neurath M.F., Pflanz S. (2013). Interleukin-33-dependent innate lymphoid cells mediate hepatic fibrosis. Immunity.

[51] Neumann K., Karimi K., Meiners J., Voetlause R., Steinmann S., Dammermann W., Lüth S., Asghari F., Wegscheid C., Horst A.K. (2017). A Proinflammatory Role of Type 2 Innate Lymphoid Cells in Murine Immune-Mediated Hepatitis. J. Immunol..

[52] Nakagawa H., Suzuki N., Hirata Y., Hikiba Y., Hayakawa Y., Kinoshita H., Ihara S., Uchino K., Nishikawa Y., Ijichi H. (2017). Biliary epithelial injury-induced regenerative response by IL-33 promotes cholangiocarcinogenesis from peribiliary glands. Proc. Natl. Acad. Sci. USA.

[53] Jeffery H.C., McDowell P., Lutz P., Wawman R.E., Roberts S., Bagnall C., Birtwistle J., Adams D.H., Oo Y.H. (2017). Human intrahepatic ILC2 are IL-13positiveamphiregulinpositiveand their frequency correlates with model of end stage liver disease score. PLoS ONE.

[54] Forkel M., Berglin L., Kekäläinen E., Carlsson A., Svedin E., Michaëlsson J., Nagasawa M., Erjefält J.S., Mori M., Flodström-Tullberg M. (2017). Composition and functionality of the intrahepatic innate lymphoid cell-compartment in human nonfibrotic and fibrotic livers. Eur. J. Immunol..

[55] Vannella K.M., Ramalingam T.R., Borthwick L.A., Barron L., Hart K.M., Thompson R.W., Kindrachuk K.N., Cheever A.W., White S., Budelsky A.L. (2016). Combinatorial targeting of TSLP, IL-25, and IL-33 in type 2 cytokine-driven inflammation and fibrosis. Sci. Transl. Med..

[56] Mjösberg J.M., Trifari S., Crellin N.K., Peters C.P., Van Drunen C.M., Piet B., Fokkens W.J., Cupedo T., Spits H. (2011). Human IL-25- and IL-33-responsive type 2 innate lymphoid cells are defined by expression of CRTH2 and CD161. Nat. Immunol..

[57] Rauber S., Luber M., Weber S., Maul L., Soare A., Wohlfahrt T., Lin N.-Y., Dietel K., Bozec A., Herrmann M. (2017). Resolution of inflammation by interleukin-9-producing type 2 innate lymphoid cells. Nat. Med..

[58] Turner J.-E., Morrison P.J., Wilhelm C., Wilson M., Ahlfors H., Renauld J.-C., Panzer U., Helmby H., Stockinger B. (2013). IL-9–mediated survival of type 2 innate lymphoid cells promotes damage control in helminth-induced lung inflammation. J. Exp. Med..

[59] Shoyab M., McDonald V.L., Bradley J.G., Todaro G.J. (1988). Amphiregulin: A bifunctional growth-modulating glycoprotein produced by the phorbol 12-myristate 13-acetate-treated human breast adenocarcinoma cell line MCF-7. Proc. Natl. Acad. Sci. USA.

[60] Busser B., Sancey L., Brambilla E., Coll J.-L., Hurbin A. (2011). The multiple roles of amphiregulin in human cancer. Biochim. Biophys. (BBA) Rev..

[61] Berasain C., García-Trevijano E.R., Castillo J., Erroba E., Lee D.C., Prieto J., Ávila M.A. (2005). Amphiregulin: An early trigger of liver regeneration in mice. Gastroenterology.

[62] Zaiss D.M., Van Loosdregt J., Gorlani A., Bekker C.P., Grone A., Sibilia M., Henegouwen P.M.P.V.B.E., Roovers R.C., Coffer P.J., Sijts A.J.A.M. (2013). Amphiregulin enhances regulatory T cell suppressive function via the epidermal growth factor receptor. Immunity.

[63] Paclik D., Stehle C., Lahmann A., Hutloff A., Romagnani C. (2015). ICOS regulates the pool of group 2 innate lymphoid cells under homeostatic and inflammatory conditions in mice. Eur. J. Immunol..

[64] Maazi H., Patel N., Sankaranarayanan I., Suzuki Y., Rigas D., Soroosh P., Freeman G.J., Sharpe A.H., Akbari O. (2015). ICOS: ICOS-Ligand interaction is required for type 2 innate lymphoid cell function, homeostasis and induction of airway hyperreactivity. Immunity.

[65] Rigas D., Lewis G., Aron J.L., Wang B., Banie H., Sankaranarayanan I., Galle-Treger L., Maazi H., Lo R., Freeman G.J. (2017). Type 2 innate lymphoid cell suppression by regulatory T cells attenuates airway hyperreactivity and requires inducible T-cell costimulator–inducible T-cell costimulator ligand interaction. J. Allergy Clin. Immunol..

[66] Sui P., Wiesner D.L., Xu J., Zhang Y., Lee J., Dyken S. (2018). Pulmonary neuroendocrine cells amplify allergic asthma responses. Science.

[67] Cardoso V., Chesné J., Ribeiro H., García-Cassani B., Carvalho T., Bouchery T., Shah K., Barbosa-Morais N.L., Harris N., Veiga-Fernandes H. (2017). Neuronal regulation of type 2 innate lymphoid cells via neuromedin U. Nature.

[68] Wallrapp A., Riesenfeld S.J., Burkett P.R., Abdulnour R.-E.E., Nyman J., Dionne D., Hofree M., Cuoco M.S., Rodman C., Farouq D. (2017). The neuropeptide NMU amplifies ILC2-driven allergic lung inflammation. Nature.

[69] Klose C.S.N., Mahlakõiv T., Moeller J.B., Rankin L.C., Flamar A.-L., Kabata H., Monticelli L.A., Moriyama S., Putzel G.G., Rakhilin N. (2017). The neuropeptide Neuromedin U stimulates innate lymphoid cells and type 2 inflammation. Nature.

[70] Nussbaum J.C., Van Dyken S.J., Von Moltke J., Cheng L.E., Mohapatra A., Molofsky A.B., Thornton E.E., Krummel M.F., Chawla A., Liang H.-E. (2013). Type 2 innate lymphoid cells control eosinophil homeostasis. Nature.

[71] Spencer S.P., Wilhelm C., Yang Q., Hall J.A., Bouladoux N., Boyd A., Nutman T.B., Urban J.F., Wang J., Ramalingam T.R. (2014). Adaptation of innate lymphoid cells to a micronutrient deficiency promotes type 2 barrier immunity. Science.

[72] Ruiter B., Patil S.U., Shreffler W.G. (2015). Vitamins A and D have antagonistic effects on expression of effector cytokines and gut-homing integrin in human innate lymphoid cells. Clin. Exp..

[73] Morita H., Kubo T., Rückert B., Ravindran A., Soyka M.B., Rinaldi A.O., Sugita K., Wawrzyniak M., Wawrzyniak P., Motomura K. (2019). Induction of human regulatory innate lymphoid cells from group 2 innate lymphoid cells by retinoic acid. J. Clin. Immunol..

[74] Laffont S., Blanquart E., Savignac M., Cénac C., Laverny G., Metzger D., Girard J.-P., Belz G.T., Pelletier L., Seillet C. (2017). Androgen signaling negatively controls group 2 innate lymphoid cells. J. Exp. Med..

[75] Johansson M.E.V., Phillipson M., Petersson J., Velcich A., Holm L., Hansson G.C. (2008). The inner of the two Muc2 mucin-dependent mucus layers in colon is devoid of bacteria. Proc. Natl. Acad. Sci. USA.

[76] Peng L., He Z., Chen W., Holzman I.R., Lin J. (2007). Effects of Butyrate on Intestinal Barrier Function in a Caco-2 Cell Monolayer Model of Intestinal Barrier. Pediatr. Res..

[77] Peng L., Li Z.-R., Green R.S., Holzman I.R., Lin J. (2009). Butyrate Enhances the Intestinal Barrier by Facilitating Tight Junction Assembly via Activation of AMP-Activated Protein Kinase in Caco-2 Cell Monolayers12. J. Nutr..

[78] Weiner H.L., da Cunha A.P., Quintana F., Wu H. (2011). Oral tolerance. Immunol. Rev..

[79] Izcue A., Coombes J.L., Powrie F. (2006). Regulatory T cells suppress systemic and mucosal immune activation to control intestinal inflammation. Immunol. Rev..

[80] Inagaki T., Moschetta A., Lee Y.-K., Peng L., Zhao G., Downes M., Yu R.T., Shelton J.M., Richardson J.A., Repa J.J. (2006). Regulation of antibacterial defense in the small intestine by the nuclear bile acid receptor. Proc. Natl. Acad. Sci. USA.

[81] Bertolino P., Trescol-Biémont M.C., Rabourdin-Combe C. (1998). Hepatocytes induce functional activation of naive CD8+T lymphocytes but fail to promote survival. Eur. J. Immunol..

[82] Herkel J., Jagemann B., Wiegard C., Garcia Lazaro J.F., Lueth S., Kanzler S., Blessing M., Schmitt E., Lohse A.W. (2003). MHC class II-expressing hepatocytes function as antigen-presenting cells and activate specific CD4 T lymphocytes. Hepatology.

[83] Burghardt S., Claass B., Erhardt A., Karimi K., Tiegs G. (2014). Hepatocytes induce Foxp3 + regulatory T cells by Notch signaling. J. Leukoc. Biol..

[84] Ebrahimkhani M.R., Mohar I., Crispe I.N. (2011). Cross-presentation of antigen by diverse subsets of murine liver cells. Hepatology.

[85] Knolle P.A., Gerken G. (2000). Local control of the immune response in the liver. Immunol. Rev..

[86] Doherty D.G. (2016). Immunity, tolerance and autoimmunity in the liver: A comprehensive review. J. Autoimmun..

[87] Milosevic I., Vujovic A., Barac A., Djelic M., Korac M., Radovanovic Spurnic A., Gmizic I., Stevanovic O., Djordjevic V., Lekic N. (2019). Gut-Liver Axis, Gut Microbiota, and Its Modulation in the Management of Liver Diseases: A Review of the Literature. Int. J. Mol. Sci..

[88] Fousekis F.S., Theopistos V.I., Mitselos I.V., Skamnelos A., Kavvadias A., Katsanos K.H., Christodoulou D.K. (2019). Specific Features of Patients with Inflammatory Bowel Disease and Primary Sclerosing Cholangitis. J. Clin. Med. Res..

[89] Kramer B., Goeser F., Lutz P., Glässner A., Boesecke C., Schwarze-Zander C., Kaczmarek D., Nischalke H.D., Branchi V., Manekeller S. (2017). Compartment-specific distribution of human intestinal innate lymphoid cells is altered in HIV patients under effective therapy. PLoS Pathog..

[90] Johansson M.E.V., Gustafsson J.K., Holmen-Larsson J., Jabbar K.S., Xia L., Xu H., Ghishan F.K., Carvalho F.A., Gewirtz A.T., Sjovall H. (2014). Bacteria penetrate the normally impenetrable inner colon mucus layer in both murine colitis models and patients with ulcerative colitis. Gut.

[91] Bergstrom K.S.B., Kissoon-Singh V., Gibson D.L., Ma C., Montero M., Sham H.P., Ryz N., Huang T., Velcich A., Finlay B.B. (2010). Muc2 Protects against Lethal Infectious Colitis by Disassociating Pathogenic and Commensal Bacteria from the Colonic Mucosa. PLoS Pathog..

[92] Camelo A., Barlow J.L., Drynan L.F., Neill D.R., Ballantyne S.J., Wong S.H., Pannell R., Gao W., Wrigley K., Sprenkle J. (2012). Blocking IL-25 signalling protects against gut inflammation in a type-2 model of colitis by suppressing nuocyte and NKT derived IL-13. J. Gastroenterol..

[93] Mankertz J., Schulzke J.-D. (2007). Altered permeability in inflammatory bowel disease: Pathophysiology and clinical implications. Curr. Opin. Gastroenterol..

[94] Huang Y., Mao K., Chen X., Sun M.A., Kawabe T., Li W., Usher N., Zhu J., Urban J.F., Paul W.E. (2018). S1P-dependent interorgan trafficking of group 2 innate lymphoid cells supports host defense. Science.

[95] Tiegs G., Hentschel J., Wendel A. (1992). A T cell-dependent experimental liver injury in mice inducible by concanavalin A. J. Clin. Investig..

[96] Louis H., Le Moine A., Flamand V., Nagy N., Quertinmont E., Paulart F., Abramowicz D., Le Moine O., Goldman M., Devière J. (2002). Critical role of interleukin 5 and eosinophils in concanavalin A–induced hepatitis in mice. Gastroenterology.

[97] Volarevic V., Mitrovic M., Milovanovic M., Zelen I., Nikolic I., Mitrovic S., Pejnovic N., Arsenijevic N., Lukic M.L. (2012). Protective role of IL-33/ST2 axis in Con A-induced hepatitis. J. Hepatol..

[98] Besnard A.-G., Guabiraba R., Niedbala W., Palomo J., Reverchon F., Shaw T.N., Couper K.N., Ryffel B., Liew F.Y. (2015). IL-33-Mediated Protection against Experimental Cerebral Malaria Is Linked to Induction of Type 2 Innate Lymphoid Cells, M2 Macrophages and Regulatory T Cells. PLoS Pathog..

[99] Martinez-Gonzalez I., Mathä L., Steer C.A., Ghaedi M., Poon G.F., Takei F. (2016). Allergen-Experienced Group 2 Innate Lymphoid Cells Acquire Memory-like Properties and Enhance Allergic Lung Inflammation. Immunity.

[100] Pellicoro A., Ramachandran P., Iredale J.P., Fallowfield J.A. (2014). Liver fibrosis and repair: Immune regulation of wound healing in a solid organ. Nat. Rev. Immunol..

[101] Perugorria M.J., Latasa M.U., Nicou A., Cartagena-Lirola H., Castillo J., Goñi S., Vespasiani-Gentilucci U., Zagami M.G., Lotersztajn S., Prieto J. (2008). The epidermal growth factor receptor ligand amphiregulin participates in the development of mouse liver fibrosis. Hepatology.

[102] McKee C., Sigala B., Soeda J., Mouralidarane A., Morgan M., Mazzoccoli G., Rappa F., Cappello F., Cabibi D., Pazienza V. (2015). Amphiregulin activates human hepatic stellate cells and is upregulated in non alcoholic steatohepatitis. Sci. Rep..

[103] Brabletz T., Kalluri R., Nieto M.A., Weinberg R.A. (2018). EMT in cancer. Nat. Rev. Cancer.

[104] Nakagawa H., Hikiba Y., Hirata Y., Font-Burgada J., Sakamoto K., Hayakawa Y., Taniguchi K., Umemura A., Kinoshita H., Sakitani K. (2014). Loss of liver E-cadherin induces sclerosing cholangitis and promotes carcinogenesis. Proc. Natl. Acad. Sci. USA.

[105] Berasain C., Castillo J., Perugorria M., Prieto J., Avila M. (2007). Amphiregulin: A new growth factor in hepatocarcinogenesis. Cancer Lett..

[106] Castillo J., Erroba E., Perugorria M.J., Santamaria M., Lee D.C., Prieto J., Ávila M.A., Berasain C. (2006). Amphiregulin Contributes to the Transformed Phenotype of Human Hepatocellular Carcinoma Cells. Cancer Res..

[107] Dai K., Huang L., Chen J., Yang L., Gong Z. (2014). Amphiregulin promotes the immunosuppressive activity of intrahepatic CD4 + regulatory T cells to impair CD8 + T cell immunity against hepatitis B virus infection. Immunology.

[108] Gasteiger G., Fan X., Dikiy S., Lee S.Y., Rudensky A.Y. (2015). Tissue residency of innate lymphoid cells in lymphoid and non-lymphoid organs. Science.

[109] Moro K., Kabata H., Tanabe M., Koga S., Takeno N., Mochizuki M., Fukunaga K., Asano K., Betsuyaku T., Koyasu S. (2016). Interferon and IL-27 antagonize the function of group 2 innate lymphoid cells and type 2 innate immune responses. Nat. Immunol..

[110] Erhardt A., Biburger M., Papadopoulos T., Tiegs G. (2007). IL-10, regulatory T cells, and Kupffer cells mediate tolerance in concanavalin A-induced liver injury in mice. Hepatology.

[111] Watanabe S., Ohnuki K., Hara Y., Ishida Y., Ikarashi Y., Ogawa S., Kishimoto H., Tanabe K., Abe R. (2010). Suppression of Con A-induced hepatitis induction in ICOS-deficient mice. Immunol. Lett..

